# Chromosome Painting in *Cercopithecus petaurista* (Schreber, 1774) Compared to Other Monkeys of the Cercopithecini Tribe (Catarrhini, Primates)

**DOI:** 10.3390/life13051203

**Published:** 2023-05-17

**Authors:** Vanessa Milioto, Luca Sineo, Francesca Dumas

**Affiliations:** Department of Scienze e Tecnologie Biologiche, Chimiche e Farmaceutiche (STEBICEF), University of Palermo, 90100 Palermo, Italy

**Keywords:** guenons, genomes, human homologies, synapomorphies

## Abstract

The Cercopithecini tribe includes terrestrial and arboreal clades whose relationships are controversial, with a high level of chromosome rearrangements. In order to provide new insights on the tribe’s phylogeny, chromosome painting, using the complete set of human syntenic probes, was performed in *Cercopithecus petaurista*, a representative species of the Cercopithecini tribe. The results show *C. petaurista* with a highly rearranged karyotype characterized by the fission of human chromosomes 1, 2, 3, 5, 6, 8, 11, and 12. These results compared with the literature data permit us to confirm the monophyly of the Cercopithecini tribe (fissions of chromosomes 5 and 6), as previously proposed by chromosomal and molecular data. Furthermore, we support the monophyly of the strictly arboreal *Cercopithecus* clade, previously proposed by the molecular approach, identifying chromosomal synapomorphies (fissions of chromosomes 1, 2, 3, 11, 12). We also add additional markers that can be useful for deciphering arboreal Cercopithecini phylogeny. For example, the fission of chromosome 8 is synapomorphy linking *C. petaurista*, *C. erythrogaster*, and *C. nictitans* among the arboreal species. Finally, a telomeric sequence probe was mapped on *C. petaurista*, showing only classic telomeric signals and giving no support to a previous hypothesis regarding a link between interspersed telomeric sequences in high rearranged genomes.

## 1. Introduction

The African Cercopithecini tribe (Gray, 1821) of the Cercopithecinae subfamily, Cercopithecidea family (Catarrhini), with numerous species and subspecies, inhabits various ecological niches which include semi-terrestrial, terrestrial, and strictly arboreal behaviors. Many researchers believe that the tribe’s species have only recently undergone rapid adaptive radiation. In fact, it is thought that the ecological and morphological diversity that characterizes this group today (with many genera and species, as well as a plethora of sub-species and geographical varieties) appeared in the late Pliocene [1,2,3]. The fact that the tribe’s radiation occurred so recently makes this group an ideal subject for evolutionary studies. Indeed, members are characterized by a high level of genetic variability and different adaptation strategies [4,5,6].

The Cercopithecini tribe, including the genera *Cercopithecus*, *Chlorocebus* (Gray 1879), *Miopithecus* (Geoffroy Saint-Hilaire 1862), *Allenopithecus* (Pocock, 1907), and *Erythrocebus* (Trouessart, 1897), is a well-known exception to Cercopithecidae’s tendency toward genomic conservatism [4,5,6,7,8,9,10].

Chromosomal analysis [5,6,7] and data from different molecular approaches [8,9,10] have led researchers to propose subdividing the tribe into two primary clades of terrestrial and strictly arboreal monkeys. However, other morphological and chromosomal evidence does not support a phylogenetically significant separation of the tribe into two subgroups [11,12,13]; for this reason, their phylogeny remains the subject of debate [14]. 

Among the terrestrial Cercopithecine tribe [8,9,10], there are species from different genera, such as *Chlorocebus aethiops* (Linnaeus, 1758) and *Erythrocebus patas* (Schreber, 1775). The arboreal clade includes the *Cercopithecus* genus, characterized by multiple speciation events which occurred during the very short period of their radiation, creating 19/25 species organized into seven or eight super species groups [2,8,9]: *C. lhoesti*, *C. cephus* (Linnaeus, 1758), *C. diana* (Linnaeus, 1758), *C. dryas* (Schwarz, 1932), *C. hamlyni* (Pocock, 1907), *C. mitis* (Wolf, 1822), *C. mona* (Schreber, 1775), and *C. neglectus* (Schlegel, 1876). With the exception of *C. lhoesti*, which is phylogenetically close to the terrestrial Cercopithecini and also considered to be in a separate genus, *Allochrocebus lhoesti* (Elliot, 1913) [3], all other species are considered to form a monophyletic arboreal clade [7,8,9,10]. The composition of the species groups, as well the relationships between arboreal species, is still under debate [2,7,8,9,10]. Many taxonomic incongruences are presumably due to incomplete lineage sorting caused by the alternative fixation/elimination of ancestral polymorphisms in populations or due to occasional introgressions via hybridization [8,15,16,17,18,19,20,21,22]. 

The cytogenetic approach, using classic analysis or molecular approaches, has been used to characterize the genomes of different and distant taxa [23,24,25,26,27]. The molecular cytogenetic approach through chromosome painting permits the identification of chromosomal homologies at the level of whole or partial chromosomes, as well as interchromosomal rearrangements (translocations, fissions, and fusions) that have occurred during genome evolution. Furthermore, chromosome painting permits the determination of chromosomal syntenies (the localization of two or more genes on the same chromosome) that have been conserved or reshaped, identifying syntenic associations in the genomes of the species being compared. Comparative chromosome banding [6] and painting [28,29] have been used to demonstrate that Catarrhini (Old World monkeys) tend to have conservative genomes. Among the conserved Old World monkeys, the genus *Cercopithecus* is one of the major exceptions, characterized instead by a notable variation in diploid number (2N = 58–72) [5,29,30,31] and a high frequency of chromosomal rearrangements and polymorphisms [4,7,14,29,30,31]. 

Despite the diversity found in the genus, only a few studies have been conducted using painting approaches on the genomic structure of *Cercopithecus* species; comparisons of chromosomal banding patterns and of whole chromosome probe “paint” mappings have permitted researchers to propose phylogenetic relationships [6,31,32,33,34,35], and to show that non-centromeric fissioning and centromere activation and deactivation [36] are noticeable chromosomal evolutionary mechanisms of the Cercopithecini tribe.

In this work, chromosome painting by fluorescence in situ hybridization (FISH) was applied to the arboreal species *Cercopithecus petaurista* (Schreber, 1774) in order to characterize its genome using whole human chromosome probes. Analyzing the results obtained in comparison with data from the literature regarding other Cercopithecini, symplesiomorphies, synapomorphies, and autapomorphies were shown. This analysis was performed in order to clarify the phylogenetic and systematic inferences regarding the tribe. Moreover, telomeric sequence probes were mapped by FISH in *C. petaurista* because recent works have suggested that interstitial telomeric sequences (ITSs) may be linked to the high rate of chromosome evolution in primates [37,38,39,40,41]. 

## 2. Materials and Methods

Skin biopsies from one male *C. petaurista* (CPE) individual were collected from the Bioparco di Sicilia (Carini, Italy). The experiments were carried out according to international ethical protocols.

Fibroblasts from skin biopsies after cell dissociation with collagenase IV (0.0186 g in 4 mL) for 1 h at 37 °C were maintained in DMEM medium (Dulbecco’s Modified Eagle medium, Sigma-Aldrich, Saint Louis, MO, USA), supplemented with antibiotics (1%) and fetal bovine serum (15%) at 37 °C for 72 h. Metaphases were obtained from fibroblast cell cultures after 1h of colcemid treatment (0.05 g/mL), followed by incubation with 0.075 M KCl (10 min at 37 °C) and Carnoy’s fixative (3 methanol:1 acetic acid) [41]. GTG-banding was produced by trypsin digestion following a previously-described protocol [38]. Chromosome-specific probes from human chromosomes (1–22 probes-XY) were kindly provided by Roscoe Stanyon and were created at the National Cancer Center (Frederick, MD, USA) after DOP-PCR. A 6MW primer was used in a secondary reaction to label the chromosomal DNA with biotin-dUTP or digoxigenin-dUTP (both from Roche) for indirect detection [42,43]. About 300–400 ng of each PCR product per probe, together with 10 μg of human Cot-1 (Invitrogen, Thermofisher scientific, Van Allen Way, Carlsbad, CA, USA) were precipitated and then dissolved in 14 μL of hybridization buffer. Probe denaturation occurred at 70 °C with 70% formamide. After FISH incubation for 72 h at 37 °C, slides were washed at low stringency conditions in the presence of 70% formamide and 2XSSC. Biotinylated DNA probes were detected with avidin coupled with fluorescein isothiocyanate (FITC, Vector Laboratories Inc., USA Ingold Road, Burlingame, CA, USA). Digoxigenin-labeled probes were detected with anti-digoxigenin antibodies conjugated with Rodamine (Roche, Eugene, OR, USA). Metaphases were counterstained with DAPI (Vectashield antifade mounting medium) [44]. 

Telomeric motif distribution was analyzed by FISH using the FITC-labeled PNA oligonucleotide probe (Panagene, Cambridge Research Biochemicals, Belasis Court, Belasis Hall Technology Park Billingham, Cleveland, UK) Hybridization was performed following the protocols furnished by Panagene, adjusting stringency conditions. The detection of the probe signals was performed at high stringency with high temperatures at 70 °C and a low saline concentrate buffer [38]. 

The metaphases were analyzed under a Zeiss Axio2 epifluorescence microscope and captured using a coupled Zeiss digital camera. The DAPI inverted karyotype was obtained using the Adobe Photoshop (CC 2022 V23.3.2) program to permit the identification of chromosomes. 

The C bands were obtained sequentially after FISH, according to a previously described protocol [45] that included denaturation with formamide. 

## 3. Results

**Figure 1:**
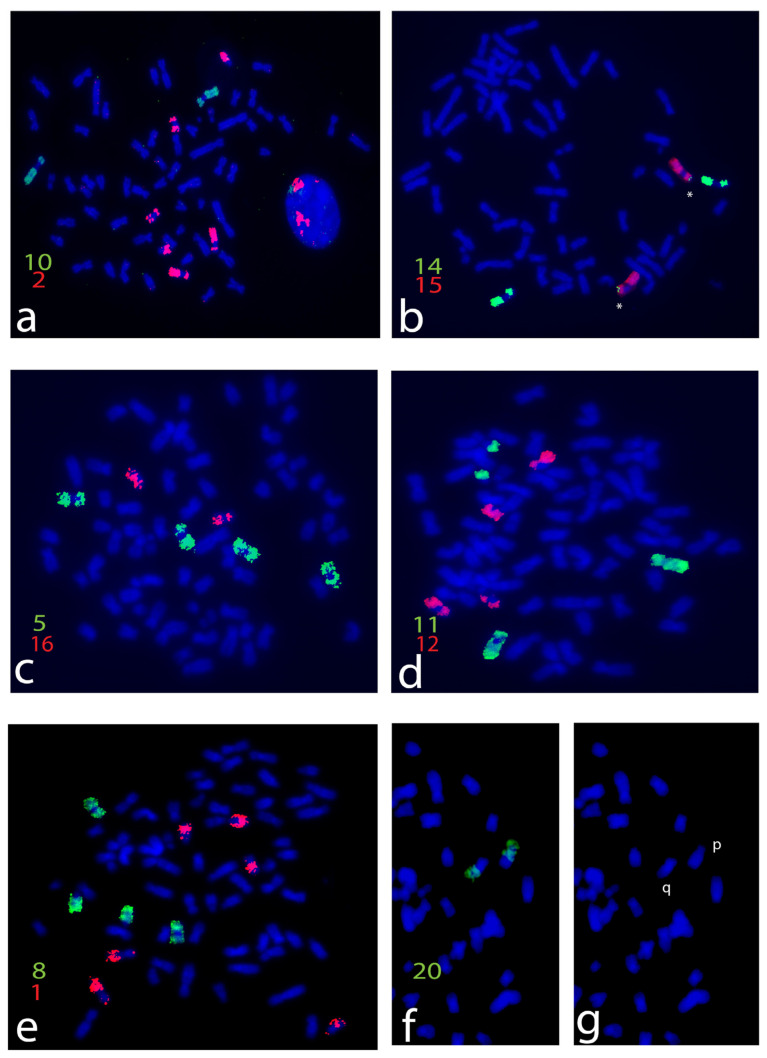
(**a**–**f**) *C. petaurista* mitotic metaphases showing examples of hybridizations of human probes. Biotin-labeled probes were detected with avidin-FITC (green) and digoxigenin-labeled probes with anti-digoxigenin-rhodamine (red). The slides were counterstained with DAPI. Human paints are indicated by numbers in green (FITC) or red (rhodamine). Note the small human syntenic association 14/15 (asterisk) (**b**) and the different pattern of synteny 20 on the two homologous chromosomes (**f**) with signals on p and q arms as it is possible to appreciate in the DAPI chromosomes (**g**). Scale bar, 5 μm.

**Figure 2:**
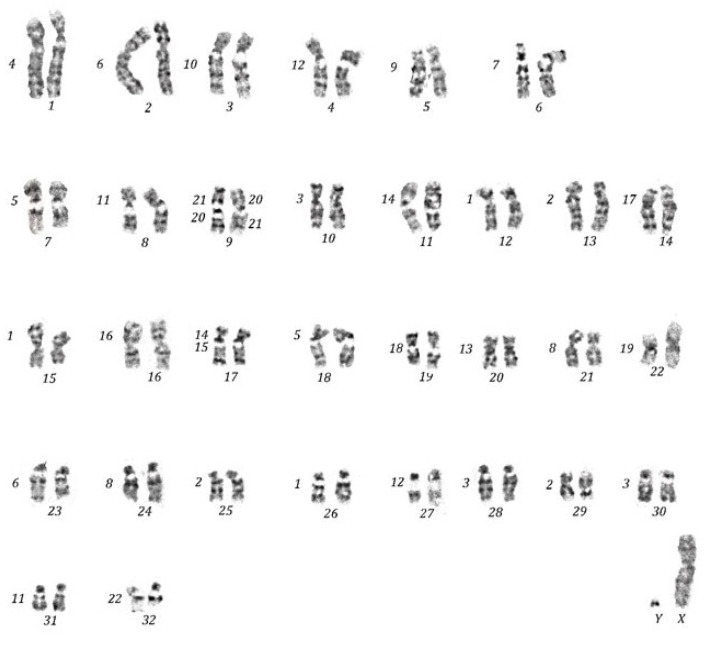
Comparative chromosome map between *C. petaurista* and human. Human syntenies are presented on the left side of each CPE chromosome on the GTG karyotype after human painting probes mapping. Human paints 20 and 21 show a heterozygous pattern.

The human probes specific for the 22 autosomes were mapped through FISH (Figure 1) on 34 homologous fragments of the haploid set of the *C. petaurista* chromosomes. The GTG banding karyotype of *C. petaurista* had 2n = 66, with 22 submetacentric/metacentric chromosome pairs (1–22) and 10 acrocentric chromosome pairs (23–32), in agreement with previous findings [30,46]. The X chromosome was the common mammalian type, and the Y chromosome was very small and acrocentric. All mapping results are indicated on the reconstructed karyotype (Figure 2) and are reported in Table 1.

The human probes showed from one to three fragments, with bright signals (Figure 1 and Figure 2). Twelve human paints (4, 7, 9, 10, 13, 15, 16, 17, 18, 19, 20, 21, 22, and X) were hybridized completely with a single *C. petaurista* chromosome. Nine human paints were found fragmented, either into two (paints 5, 6, 8, 11, 12, and 14) or three segments (paints 1, 2, and 3) (Figure 1 and Figure 2). 

**Figure 3:**
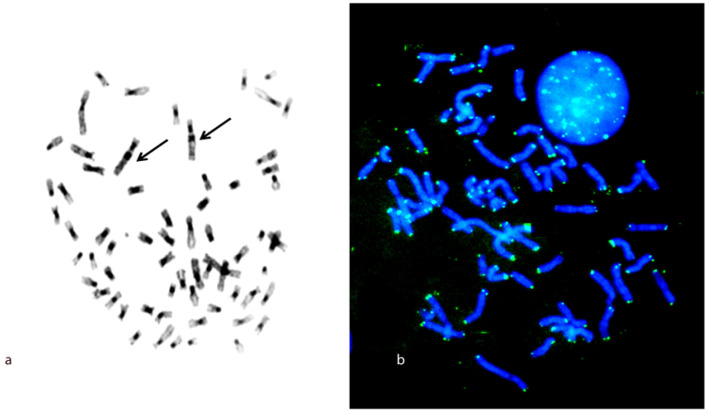
*C. petaurista* metaphase with large C bands at centromeres of all chromosomes plus an additional interstitial signal, indicated with arrows (**a**), and telomeric probe signals labeled with FITC (green), (**b**), Scale bar, 5 μm.

Human probe 14 covers *C. petaurista* chromosome 11, and a very small segment falls on chromosome 17 in association with synteny 15 (Figure 1b and Figure 2). Human probes 20/21 both fall on the same chromosome pair 9, showing a heterozygous pattern (Figure 1f,g and Figure 2). C banding obtained after FISH showed large C bands at the centromeres of all chromosome pairs. An additional sizeable interstitial band below the centromere was present on a large submetacentric chromosome pair in *C. petaurista* (Figure 3a). The telomeric probes gave signals at the terminal positions of all chromosome pairs (Figure 3b). 

## 4. Discussion

The Cercopithecini tribe, with its chromosomal variability distributed across its different species and genera, is a prime example of genome evolution related to niche separation and geographical barriers which has been scarcely explored. Cytogenetic and molecular approaches have supported clustering the tribe into two groups: primarily terrestrial and more chromosomally conservative monkeys, and arboreal and chromosomally non-conservative guenons. However, the composition of these species groups, and the relationships between the arboreal species are still unclear [7,8,9,10,11,12,13]. In order to acquire further information regarding the complex phylogenomics of this tribe, we utilized the painting approach, using the complete set of human probes on *C. petaurista*. *C. petaurista* is characterized by a forest ecology and a rather rearranged karyotype, with a diploid number of 2n = 66. The sample analyzed here confirms the species’ diploid number and the GTG-banding pattern (Figure 2), in agreement with what has previously been described in the literature [6,30,46]. 

Although the species of the tribe analyzed showed a high level of rearrangements when compared to the generally well-conserved Old World monkeys, only a few species have been analyzed so far through the molecular cytogenetic approach [7,31,34,35]. The molecular cytogenetic analyses through paint mapping allowed the effective detection of chromosomes homologous to human chromosomes and of rearrangements between mammal taxa. From these analyses, hypothetical ancestral karyotypes of the major nodes of the mammalian phylogenetic tree have been proposed [47,48]. The painting data here obtained on *C. petaurista* were compared with these hypothetical karyotypes (Ancestral Primate Karyotype APK 2n = 50 1, 2pq, 2q, 3/21, 7b, 7a/16, 10a, 10q, 12/22 x2, 14/15, 16q, 19p, 19q; Ancestral Catarrhini Karyotype ACK 2n = 46, fusion of chromosomes 7, 10, 16, fission 3/21, 2a, 2b, 14/15) and with chromosomal painting data from both terrestrial and arboreal Cercopithecine species, paying particular attention to the latter (Table 1).

The ancestral mammalian syntenic association 3/21 was not found in *C. petaurista*, as in all Cercopithecini species and in the ancestral Catarrhine karyotype [47]. Indeed, human probes 3 and 21 were mapped onto different chromosomes (Figure 2).

The ancestral mammal syntenic association 14/15 was fissioned in the ancestral Catarrhine karyotype [47], but is still present in the Cercopithecine species. In *C. petaurista*, human probes 14 and 15 covered two different bi-armed chromosomes, 11 and 17, respectively (Figure 1b and Figure 2), and the 14/15 association does not seem to be present. However, on chromosome 17, a very small and hard to detect fragment of synteny 14 was found in association with synteny 15 (Figure 1b). This evidence is supported by what is shown in the other species here analyzed, where the 14/15 syntenic association is present and human probe 14 is split into two fragments. The different size of human synteny 14 (in association with synteny 15) in the species analyzed indicates that the fissions presumably occurred with different breakpoints and are consistent with the high level of rearrangements previously shown in other primate taxa [49,50]. Human probe 15 can also be found in a single block or split into two blocks in the analyzed taxa (Table 1). Thus, human chromosomes 14 and 15 have been subject to different chromosomal rearrangements involving different breakpoints, giving different fragments [49,50,51]. It would be useful in the future to verify the breakpoints of these syntenies in the Cercopithecini species using the BAC (bacterial artificial chromosomes) mapping approach, which can provide new insight into their chromosomal evolution [52].

The 20/21 syntenic association is an apomorphic trait of all Cercopithecini species (Table 1), also present as a polymorphism known as a “tribal-specific” polymorphism [14]. In *C. petaurista*, the 20/21 syntenic association is found on chromosome 9 in heterozygosity, as shown here through the paint patterns (Figure 1f,g and Figure 2) and in agreement with previous banding, painting [4,5], and BAC [33] data mapping. 

In all Cercopithecine species, including *C. petaurista* and the ancestral Catarrhine karyotype [47], human chromosomes 9, 10, 13, 16–19, and 20–22 are homologous to just one chromosome (Figure 1 and Figure 2). A two-fragment split of human chromosome 10 homologue has been found as an autapomorphy only in *Cercopithecus campbelli* (Waterhouse, 1838), where only a few painting probes have been previously mapped [31].

**Figure 4:**
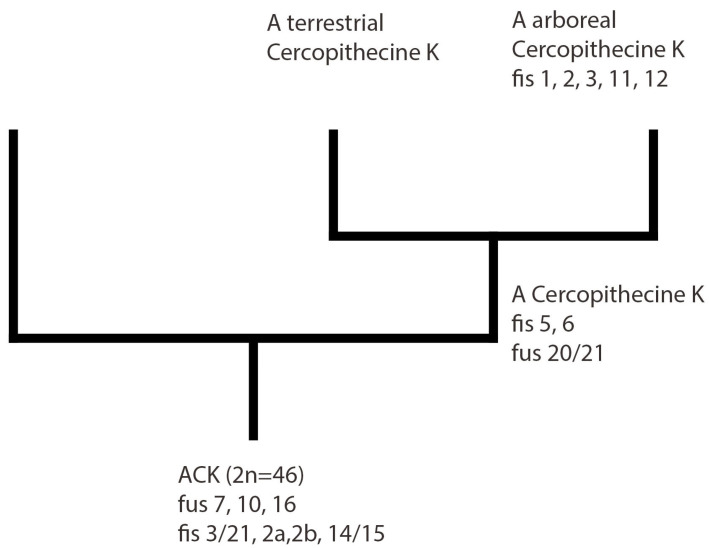
Chromosome painting data (with references to human chromosomes and associations) are reported on the molecular phylogenetic tree of the Cercopithecine tribe, here redraw according a previous reconstruction [7]. Ancestral Catarrhini Karyotype (ACK). Putative synapomorphies are mapped in the nodes for the Ancestral Cercopithecini tribe (A Cercopithecini K) and for the arboreal clade.

Human probes 1, 2, 3, 5, 6, 8, 11, 12 show from two to three fragments in *C. petaurista*; the rearrangements affecting these syntenies represent symplesiomorphies or synapomorphies linking either all members of the Cercopithecini tribe or the arboreal clade, or just a few species. These data support the previously hypothesized molecular phylogenetic groups [8] (Figure 4). In particular, human probes 1, 2, and 3 are fragmented into at least three fragments in the species of the arboreal clade (three segments in *C. petaurista*, *C. erythrogaster*, and *C. nictitans stamptii*, four fragments in *C. wolfi* and *C. neglectus*), but only one or two fragments are present in the terrestrial Cercopithecini *C. aethiops* and *Erythrocebus patas* (Table 1). Analyzing this evidence, it could be hypothesized that having at least three fragments of the two syntenies is a feature common to the arboreal clade (Figure 4). 

Human paints 5 and 6 in *C. petaurista* are fissioned into two fragments, in agreement with what has been shown in other Cercopithecini [7,31]. These features represent chromosomal symplesiomorphies linking all the Cercopithecine clade and supporting the monophyly of the tribe (Figure 4).

Human synteny 8 is in one segment in the ancestral Catarrhine karyotype, as well as in the other Cercopithecini, except for *C. petaurista*, *C. erythrogaster*, and *C. nictitans*, where it is in two segments. The presence of two segments of synteny 8 is a synapomorphy linking these latter three arboreal species. Only in *C. neglectus* is there an association of human syntenies 1/8, a derived feature considered to be an autapomorphy (Table 1). 

Human probes 11 and 12 are split into two fragments in *C. petaurista* (Figure 1d and Figure 2), as well in all the arboreal species analyzed here, except for *C. neglectus* (Table 1). These two human chromosomes are present as a single fragment in the ancestral Catarrhine karyotype [47] as well as in other Cercopithecini species (Table 1). From this analysis, it can be hypothesized that the splitting of these syntenies into two segments can be considered a feature linking all species of the arboreal clade (Figure 4).

On the other hand, human probes 4 and 7 are present as single fragments in *C. petaurista* but in diverse conditions in other taxa. Human synteny 4 is conserved as one segment in some arboreal species, such as *C. erythrogaster* and *C. neglectus*, and in the terrestrial *Erytrocebus patas*, as well as in the ancestral Catarrhine karyotype. It is fragmented into two or three segments in the other arboreal species, for example, *C. nictititans* and *C. wolfi*, and the terrestrial *Chlorocebus aethiops*. Human synteny 7 is present as one segment in the arboreal *C. petaurista*, *C. erytrogaster*, and *C. neglectus*, as also occurs in the ancestral Catarrhine karyotype [47]; however, it is split into two fragments in other Cercopithecini, both arboreal and terrestrial, such as *C. wolfi*, *C. nictititans*, and *Erythrocebus patas*, and *C. aethiops*, respectively. The complex conditions of human syntenies 4 and 7 can be explained as the consequence of a presumed presence of polymorphic forms in the ancestral Cercopithecini group before the splitting of the terrestrial and arboreal clades. Fissions of human syntenies 4 and 7 are not phylogenetically informative but may support the hypothesis regarding the occurrence of polymorphisms among *Cercopithecus* species [4,7]. In any case, it cannot be excluded that this complex condition is the result of convergent evolution [53]. 

Despite its highly rearranged karyotype characterized by many fissions, telomeric probe mapping on *C. petaurista* showed only classic telomeric signals; indeed, no ITSs far from the chromosomal ends were found (Figure 3). The hypothesis that non-centromeric fissions could have been accompanied by ITS formation in Cercopithecidae [37] is not supported by this evidence, though it is not possible to exclude the possibility that lowly amplified sequences could be present but undetectable at this level of resolution. 

## 5. Conclusions

The data shown here confirm the monophyly of the Cercopithecini tribe as well as of the arboreal clade, and add additional markers that can be useful for deciphering Cercopithecini phylogeny, especially the relationships between arboreal species. The comparison of *C. petaurista* with other previously analyzed species permits us to confirm that fissions of homologues to human chromosomes 5 and 6 are symplesiomorphies linking all Cercopithecini, in agreement with the previous molecular proposed phylogeny [7,8,9,10]. Furthermore, the comparative analysis shows that human chromosome 2, 3, 11, and 12 homologue fissions are chromosomal synapomorphies, supporting the previously proposed molecular phylogenetic arboreal clade [8]; in addition, the fission of homologues to human chromosome 8 is a synapomorphy linking *C. petaurista*, *C. erythrogaster*, and *C. nictitans* among the arboreal species. Further analyses with a multidisciplinary approach and a wider range of samples are needed to clarify the relationships between the species of the arboreal clade.

## Figures and Tables

**Table 1:** Number of hybridization signals revealed by human chromosome paints on the autosomal haploid chromosome complement of the various species of the Cercopithecini tribe previously analyzed through painting. The presence of syntenic associations is indicated with +. The terrestrial species of the Cercopithecine tribe previously studied through painting are *Erythrocebus patas*—EPA (2n = 54) and *Chlorocebus aethiops*—CAE (2n = 60) [7,34]. The arboreal species previously studied through painting are *Cercopithecus wolfi*—CWO (Meyer, 1891) (2n = 72) [35], *Cercopithecus neglectus*—CNE (Schlegel, 1876) (2n = 62) [7], *Cercopithecus erythrogaster*—CER (Gray, 1866) (2n = 66), and *Cercopithecus nictitans stamptii*—CNI (Linnaeus, 1766) (2n = 70) [31]. *Cercopithecus petaurista*—CPE data are from this paper.

Species	EPA (2n = 54)	CAE (2n = 60)	CPE (2n = 66)	CER (2n = 66)	CNE (2n = 62)	CNI (2n = 70)	CWO (2n = 72)
HSA paints							
1	1	2	3	3	4	3	4
2	2	2	3	3	3	3	3
3	2	2	3	3	3	3	3
4	1	2	1	1	1	2	3
5	2	2	2	2	2	2	2
6	2	2	2	2	2	2	2
7	2	2	1	1	1	2	2
8	1	1	2	2	1 (with 1)	2	1
9	1	1	1	1	1	1	1
10	1	1	1	1	1	1	1
11	1	1	2	2	1	2	2
12	1	1	2	2	1	2	2
13	1	1	1	1	1	1	1
14	1	2	2	2	2	2	2
14/15	+	+	+	+	+	+	+
15	2	2	1	1	2	1	1
16	1	1	1	1	1	1	1
17	1	1	1	1	1	1	1
18	1	1	1	1	1	1	1
19	1	1	1	1	1	1	1
20	1	1	1	1	1	1	1
20/21	+	+	+	+	+	+	+
21	1	1	1	1	1	1	1
22	1	1	1	1	1	1	1

## Data Availability

All data generated or analyzed during this study are included in this article.
